# Functional outcomes following knee replacement in community-dwelling older adults

**DOI:** 10.1186/s12877-023-03925-y

**Published:** 2023-05-02

**Authors:** Yuanyuan Wang, Alice Owen, Angus Franks, Ilana Ackerman, Sharyn M. Fitzgerald, Susan Liew, Robyn L. Woods, Anita E. Wluka, John J. McNeil, Flavia M. Cicuttini

**Affiliations:** 1 School of Public Health and Preventive Medicine, Monash University, 553 St Kilda Road, Melbourne, VIC 3004 Australia; 2 Alfred Hospital, Melbourne, Australia

**Keywords:** Physical function, Knee replacement, Older adults

## Abstract

**Background:**

Knee replacements are increasingly performed in older adults but uncertainty remains regarding their benefits in the context of age-related decline in physical function and other comorbidities. This study aimed to examine (1) the effect of knee replacement on functional outcomes in the context of age-related decline in physical function and (2) the factors associated with minimal important improvement in physical function after knee replacement in community-dwelling older adults aged ≥ 70 years.

**Methods:**

This cohort study was performed within the ASPREE trial, with 889 participants undergoing knee replacement during the trial and 858 age- and sex-matched controls without knee or hip replacement identified from 16,703 Australian participants aged ≥ 70 years. Health-related quality of life was assessed annually using the SF-12, including its physical and mental component summary (PCS and MCS). Gait speed was measured biennially. Multiple linear regression and analysis of covariance were used to adjust for potential confounders.

**Results:**

Participants with knee replacement had significantly lower pre- and post-replacement PCS scores and gait speed compared with age- and sex-matched controls. Participants with knee replacement had significant improvement in PCS score following knee replacement (mean change 3.6, 95% CI 2.9–4.3) while PCS score remaining unchanged in age- and sex-matched controls (-0.02, 95% CI -0.6 to 0.6) during follow-up period. The greatest improvements were observed for bodily pain and physical function. Following knee replacement, 53% of participants experienced minimal important improvement in PCS score (increased by ≥ 2.7), while 24% experienced worsened PCS score (reduced by > 2.7). Participants experiencing improved PCS score postoperatively had significantly lower PCS and higher MCS scores pre-surgery.

**Conclusions:**

Although community-based older adults experienced a significant improvement in PCS scores after knee replacement, their postoperative physical functional status remained significantly lower than age- and sex-matched controls. The degree of preoperative physical function impairment was a strong predictor of functional improvement, suggesting that this could be an important consideration when identifying older people most likely to benefit from knee replacement surgery.

**Supplementary Information:**

The online version contains supplementary material available at 10.1186/s12877-023-03925-y.

## Background

Knee osteoarthritis is common with increasing age, causing pain and functional limitation [1]. Total knee replacement (TKR) has been shown to be a cost-effective intervention for severe osteoarthritis which relieves symptoms and restores function [2]. Previous studies have consistently demonstrated significant benefits of TKR on improving function and quality of life in hospital and arthroplasty registry settings [3–7]. However, 20–30% of patients continue to experience pain and functional limitation after total joint replacement [8], and up to one quarter of TKR recipients are dissatisfied with their outcomes despite undergoing a technically successful procedure [9, 10]. Based on data from different countries, the median age at TKR was 69–70 years old [11–14], which means that almost half of all TKRs for osteoarthritis were performed in older adults aged 70 years and over. A recent systematic review showed improvements of pain, function, and quality of life following TKR in patients aged 65 years and over [15]. However, there remains significant uncertainty regarding the effectiveness of knee replacement in older adults who also experience age-related decline in physical function compounded by comorbidities. To date there are no data in older adults on how much TKR would improve physical functional status towards the comparable level of their age- and sex-matched peers. For older adults living with osteoarthritis and often a greater burden of comorbidity, it is important to identify the factors associated with clinically important improvement in physical function postoperatively because there is the potential that a knee replacement may have modest benefits in this context. Such evidence is needed to inform clinical decision making around a major surgical intervention that is costly and entails a lengthy period of recovery, but has the potential to substantially improve physical function, quality of life and independence.

Thus, the aim of our study was (1) to examine the effect of knee replacement on functional outcomes in older adults aged ≥ 70 years, compared to age- and sex-matched controls without joint replacement; and (2) to identify the factors associated with minimal important improvement in physical function in participants who had a knee replacement. This study was embedded within a large community-based clinical trial of healthy community-dwelling older adults aged ≥ 70 years. This approach enables the impact of knee replacement in older people to be examined in the context of age-related decline in physical function, which increases after the age of 70 years [16, 17].

## Methods

### Study population and setting

This cohort study was performed within the ASPirin in Reducing Events in the Elderly (ASPREE) trial [18, 19], a randomised, placebo-controlled trial determining whether 100 mg/d aspirin extended disability-free survival in 19,114 healthy, community-dwelling individuals from Australia (aged ≥ 70 years) and the USA (aged ≥ 65 years). The ASPREE study was conducted in accordance with the Declaration of Helsinki 1964 as revised in 2008, the NHMRC Guidelines on Human Experimentation, the federal patient privacy (HIPAA) law and ICH-GCP guidelines and the International Conference of Harmonisation Guidelines for Good Clinical Practice. ASPREE exclusion criteria included history of diagnosed cardiovascular events, dementia or presence of physical disability (major difficulty with performing independently any one of six basic activities of daily living), resulting in a relatively ‘healthy’, independently-living cohort at enrolment. The study was approved by Ethics Committees in Australia and the USA, and registered on Clinicaltrials.gov (NCT01038583, registered 24/12/2009). All participants provided written informed consent. The median follow-up period was 4.7 years. The sample for the present study was drawn from the 16,703 Australian participants. The study was approved by site-specific ethics committees including the Royal Australasian College of General Practitioners Ethics Committee (NREEC 02/22b), the Monash University Human Research Ethics Committee (2006/745MC), the Human Research Ethics Committee (Tasmanian) Network (H0008933), ACT Health Human Research Ethics Committee (11/07.997), and The University of Adelaide Human Research Ethics Committee (H-250–2011). Australia has a universal healthcare system providing publicly-funded access to joint replacement, with subsidised access available through private health providers.

### Identification of participants with knee replacement and controls

**Fig. 1:**
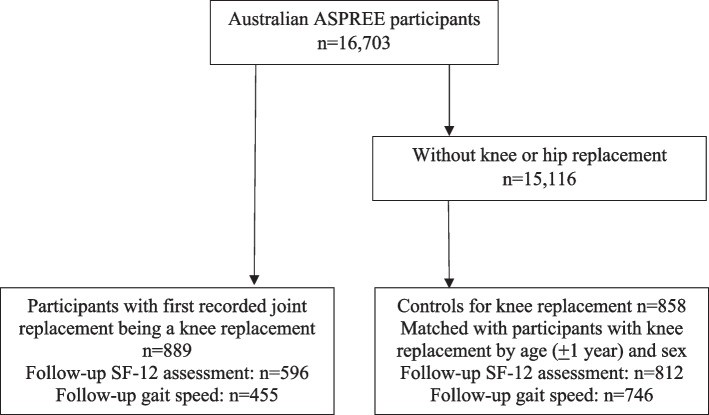
Participants with knee replacement and age- and sex-matched controls

Clinical documentation relating to hospitalisations for knee and hip joint surgical procedures during the ASPREE trial was reviewed, and participants with any knee replacement procedure (> 95% with the indication recorded as osteoarthritis) were identified. The first recorded in-trial knee replacement was used to confer status as having a knee replacement. Participants with knee replacement were matched 1:1 by age at time of knee replacement (± 1 year) and sex with controls (age at study entry) without knee or hip replacement during the trial (Fig. 1). Baseline was defined as the pre-surgery study visit for participants with knee replacement and the ASPREE baseline study visit for controls.

### Outcome measures

Health-related quality of life was assessed annually using the Medical Outcomes Study Short Form 12 Health Survey Version 2 (SF-12v2) [20], a generic health profile instrument consisting of 12 items that measure 8 health domains to assess physical and mental health: physical functioning, role physical, bodily pain, general health, vitality, social functioning, role emotional, and mental health. The scores of these domains can be weighted and summarised into two composite scores: physical component summary (PCS) score and mental component summary (MCS) score [21]. The scores of PCS, MCS, and the 8 domains range 0–100 with higher values indicating better health status [21]. This scale has been used in older people undergoing TKR [22, 23].

Gait speed, a simple, objective indicator of physical function and mobility [24, 25], was measured at the randomisation visit and thereafter biennially, as the time in seconds to walk 3 m at participant’s usual walking pace from a standing start, with a gait aid, if used. Time on the faster of two walks was the final gait speed measure [26].

For participants with knee replacement, the pre-knee replacement measure was selected as the study visit assessment undertaken immediately prior to the hospitalisation (maximum 12 months from study visit to hospitalisation); the post-knee replacement measure was taken as the first available study visit assessment at least 6 months after the hospitalisation. For controls, outcomes were examined at study baseline, and then at 1 year for physical and mental health and 2 years for gait speed after randomisation.

### Covariates

Weight and height were measured, and data on date of birth, sex, years of education, and comorbidities (diabetes, dyslipidemia, hypertension, chronic kidney disease, and cancer) were collected using questionnaires as previously described [18]. Body mass index (BMI) was calculated from weight/height^2^. Self-reported history of joint replacement prior to study enrolment was obtained from a questionnaire in a sub-study, the ASPREE Longitudinal Study of Older Persons [27].

### Statistical analysis

Characteristics of participants with knee replacement and age- and sex-matched controls were compared using independent samples t-tests or chi-square tests, when appropriate. For aim 1 comparisons of changes in physical and mental health and gait speed between participants with knee replacement and controls, independent samples t-tests were used for unadjusted analyses, and multiple linear regression models were fitted with knee replacement vs control as the independent variable and change in functional outcomes as the dependent variable, adjusted for baseline BMI and time between outcome measures. For aim 2, participants with knee replacement were categorised into three groups based on the minimal important change in PCS score which was 2.7 points after TKR [28]: worsened (> 2.7 reduction), stable (change ≥ -2.7 and < 2.7), and improved (≥ 2.7 increase) physical function. To compare participant characteristics among the physical function categories, analysis of variance (ANOVA) was used for unadjusted analyses and analysis of covariance (ANCOVA) was used to adjust for covariates.

Stratified analyses by age category and sex were performed. Sensitivity analyses were undertaken excluding participants who self-reported a history of joint replacement at baseline. Additional adjustment for education was performed. To reduce the risk of type 1 error, adjustment for multiple comparisons was undertaken and alpha set at 0.005 for this study. All analyses were undertaken using STATA 16 (College Station, Texas USA).

## Results

There were 889 participants with knee replacement and 858 age- and sex-matched controls (Fig. 1). Participant characteristics at ASPREE baseline are presented in Table 1. Participants with knee replacement had significantly higher BMI and lower PCS score than controls. The scores of physical function, role physical, bodily pain, general health, vitality, and mental health were significantly lower in participants with knee replacement compared with controls. Presence of comorbidities, education, MCS score, scores of social functioning and role emotional, and gait speed did not differ significantly between participants with knee replacement and controls. The median time between baseline and follow-up outcome measures was 464 days (range 238–1115) for participants with knee replacement and 361 days (range 236–716) for controls.

**Table 1:** Characteristics of participants with knee replacement and matched controls at ASPREE baseline

	**Participants with knee replacement** ***n* = 889**	**Age- and sex-matched controls** ***n* = 858**	***P***
Age (matched), years	77.4 (4.2)	77.4 (4.2)	0.94
Females, n (%)	545 (61.3)	529 (61.7)	0.88
Education > 12 years, n (%)	323 (36.3)	357 (41.6)	0.02
Body mass index, kg/m^2^	29.9 (4.9)	27.8 (4.4)	< 0.0001
Diabetes, n (%)	85 (9.6)	83 (9.7)	0.94
Dyslipidemia, n (%)	626 (70.4)	584 (68.1)	0.29
Hypertension, n (%)	694 (78.1)	652 (76.0)	0.30
Chronic kidney disease, n (%)	208 (25.3)	224 (28.3)	0.18
Cancer, n (%)	166 (18.7)	164 (19.1)	0.35
Physical component summary score of SF-12	43.9 (9.6)	47.6 (9.0)	< 0.0001
Mental component summary score of SF-12	56.0 (7.6)	55.8 (7.0)	0.59
Physical function	43.5 (10.4)	47.3 (9.6)	< 0.0001
Role physical	47.1 (9.2)	49.5 (8.3)	< 0.0001
Bodily pain	44.9 (10.5)	49.2 (10.1)	< 0.0001
General health	50.4 (8.0)	51.8 (7.6)	0.0003
Vitality	53.5 (8.5)	54.8 (8.3)	0.001
Social functioning	52.8 (7.6)	53.5 (7.3)	0.04
Role emotional	50.7 (8.1)	51.4 (7.2)	0.049
Mental health	53.7 (8.3)	54.9 (7.9)	0.002
Gait speed, m/sec	1.02 (0.22)	1.03 (0.23)	0.55

Data presented as mean (standard deviation) for continuous variables, and no (%) for categorical variables

### Knee replacement and PCS

Prior to knee replacement, participants had significantly lower PCS score than age- and sex-matched controls (40.4 vs 47.7, *p* < 0.0001) (Table 2; Supplementary Fig. 1 A). There was a significant improvement in PCS score following knee replacement (mean change 3.7, standard deviation [SD] 10.1), while no significant change was observed for controls over that time (-0.1, SD 7.5). Post-knee replacement PCS score remained significantly lower than for controls (44.1 vs 47.6, *p* < 0.0001) (Table 2; Supplementary Fig. 1 A). In analyses adjusted for BMI and time between outcome measures, significant improvement in PCS score was observed in participants following knee replacement but not in controls (mean change 3.6 vs -0.02), with a significant between-group difference (3.6, 95% confidence interval [CI] 2.7, 4.6) (Table 2).

**Table 2:** Baseline and follow-up measures and change in physical and mental health and gait speed in participants with knee replacement and controls

	**Participants with knee replacement** ***N* = 596**			**Age- and sex-matched controls** ***N* = 812**			**Difference between groups**	
	Baseline Mean (SD)	Follow-up Mean (SD)	Change^b^ Mean (95% CI)	Baseline Mean (SD)	Follow-up Mean (SD)	Change^b^ Mean (95% CI)	Change^b^ Mean (95% CI)	*P*
PCS	40.4 (10.6)	44.1 (10.3)	3.6 (2.9, 4.3)	47.7 (8.9)	47.6 (9.3)	-0.02 (-0.6, 0.6)	3.6 (2.7, 4.6)	< 0.001
MCS	56.7 (8.3)	55.8 (8.1)	-1.0 (-1.6, -0.3)	55.9 (7.0)	55.8 (7.1)	0.02 (-0.6, 0.6)	-1.0 (-1.8, -0.1)	0.02
Physical function	40.6 (11.3)	44.0 (11.3)	3.4 (2.6, 4.2)	47.4 (9.4)	47.4 (10.2)	0.2 (-0.5, 0.9)	3.1 (2.1, 4.2)	< 0.001
Role physical	45.0 (10.0)	46.6 (9.4)	1.4 (0.6, 2.2)	49.6 (8.2)	49.5 (8.7)	0.1 (-0.6, 0.7)	1.3 (0.3, 2.3)	0.01
Bodily pain	41.1 (11.1)	46.2 (11.1)	4.9 (3.9, 5.8)	49.3 (9.9)	49.3 (10.1)	-0.02 (-0.8, 0.7)	4.9 (3.7, 6.1)	< 0.001
General health	49.4 (9.1)	50.0 (8.8)	0.6 (-0.004, 1.3)	51.9 (7.4)	51.6 (8.6)	-0.3 (-0.9, 0.2)	1.0 (0.1, 1.8)	0.03
Vitality	52.1 (9.4)	52.9 (9.2)	0.6 (-0.1, 1.4)	54.8 (8.3)	54.6 (8.4)	-0.1 (-0.7, 0.5)	0.7 (-0.2, 1.7)	0.15
Social functioning	51.7 (8.8)	52.5 (8.3)	0.8 (0.03, 1.5)	53.5 (7.2)	53.3 (7.4)	-0.2 (-0.8, 0.5)	0.9 (-0.05, 1.9)	0.06
Role emotional	50.2 (8.8)	50.5 (8.8)	0.3 (-0.5, 1.0)	51.5 (7.1)	51.6 (7.6)	0.2 (-0.3, 0.8)	0.001 (-0.9, 0.9)	1.00
Mental health	54.2 (8.3)	54.3 (8.2)	-0.005 (-0.7, 0.7)	55.0 (7.9)	55.0 (7.8)	0.1 (-0.5, 0.6)	-0.1 (-1.0, 0.8)	0.86
Gait speed (m/sec)^a^	0.97 (0.21)	0.93 (0.20)	-0.04 (-0.06, -0.02)	1.04 (0.22)	0.99 (0.22)	-0.05 (-0.06, -0.04)	0.01 (-0.01, 0.03)	0.29

*SD* Standard deviation, *CI* Confidence interval, *PCS* Physical component summary score of SF-12, *MCS* Mental component summary score of SF-12

^a^ *n* = 455 for participants with knee replacement and *n* = 746 for controls

^b^ Adjusted for baseline body mass index and days between outcome measures

### Knee replacement and MCS

Participants had a slightly higher MCS score prior to knee replacement than age- and sex-matched controls (56.7 vs 55.9, *p* = 0.04) (Table 2; Supplementary Fig. 1 B). There was a decline in MCS score following knee replacement (mean change -0.9, SD 8.5) which was not observed in controls (-0.1, SD 7.0), resulting in very similar MCS score at follow-up (55.8 vs 55.8, *p* = 0.94) (Table 2; Supplementary Fig. 1 B). In adjusted analyses, participants with knee replacement had a decline in MCS score which was not seen in controls (mean change -1.0 vs 0.02), with little between-group difference (-1.0, 95% CI -1.8, -0.1) (Table 2).

### Knee replacement and SF-12 health domains

Prior to knee replacement, participants had significantly lower scores than age- and sex-matched controls in physical function, role physical, bodily pain, general health, vitality, social functioning, and role emotional (Table 2; Supplementary Fig. 1 C-J). Participants continued to have significantly lower scores after knee replacement than controls in physical function, role physical, bodily pain, general health, and vitality (all *p* ≤ 0.001) (Table 2; Supplementary Fig. 1 C-J). In adjusted analyses, participants had significant improvement in physical function, role physical, and bodily pain following knee replacement, while controls experienced no significant change in any domains. Between-group differences were significant for physical function and bodily pain (Table 2).

### Knee replacement and gait speed

Gait speed was significantly lower in participants pre-knee replacement compared with age- and sex-matched controls (0.97 vs 1.04 m/s, *p* < 0.0001) (Table 2). Gait speed declined in both participants with knee replacement (mean change -0.04 m/s, SD 0.18) and controls (-0.05 m/s, SD 0.18) of a similar magnitude over the follow-up period. Post-knee replacement gait speed remained significantly lower than that of controls (0.93 vs 0.99 m/s, *p* < 0.0001) (Table 2). In adjusted analyses, participants with knee replacement and controls experienced declines in gait speed of a similar magnitude (mean change -0.04 m/s vs -0.05 m/s), with no between-group difference (0.01, 95% CI -0.01, 0.03) (Table 2).

Similar results were observed for males and females, with male participants experiencing a greater improvement than female participants postoperatively in PCS (4.2 vs 3.2), physical function (4.5 vs 2.2), and bodily pain (6.0 vs 4.0) (Supplementary Tables 1 and 2). Similar results were also seen in analyses additionally adjusted for education, or stratified by age category. The results did not change in sensitivity analyses excluding participants with self-reported joint replacement before ASPREE trial (Supplementary Table 3).

### Factors for minimal important change in PCS score after knee replacement

Baseline characteristics of participants with knee replacement were compared among groups with worsened, stable, and improved PCS score based on the minimal important change (Table 3). Following knee replacement, 316 (53%) participants experienced improved PCS score, and 145 (24%) experienced worsened PCS score. Participants who experienced improved PCS score were slightly younger, had significantly lower preoperative scores of PCS, physical function, role physical, bodily pain, general health, and vitality, and significantly higher preoperative MCS score. There were no significant differences among the PCS status groups in sex, education, BMI, presence of comorbidities, scores of social functioning, role emotional, and mental health, or gait speed. Similar results were observed when adjustment for these covariates was performed.

**Table 3:** Baseline characteristics of participants with knee replacement based on minimal important change in self-reported physical function

	**Worsened PCS** **(PCS change < -2.7)** ***n* = 145**	**Stable PCS** **(PCS change -2.7 to 2.7)** ***n* = 135**	**Improved PCS** **(PCS change ≥ 2.7)** ***n* = 316**	***P***
Age at joint replacement, years	77.6 (4.8)	77.1 (3.8)	76.5 (3.9)	0.02
< 75 years, n (%)	53 (36.6)	50 (37.0)	139 (44.0)	0.20
≥ 75 years, n (%)	92 (63.4)	85 (63.0)	177 (56.0)	
Females, n (%)	92 (63.5)	76 (56.3)	181 (57.3)	0.38
Education > 12 years, n (%)	52 (35.9)	41 (30.4)	117 (37.0)	0.39
Body mass index, kg/m^2^	30.1 (5.1)	29.7 (4.7)	30.0 (4.9)	0.77
Diabetes, n (%)	19 (13.1)	12 (8.9)	29 (9.2)	0.38
Dyslipidemia, n (%)	99 (68.3)	88 (65.2)	228 (72.2)	0.31
Hypertension, n (%)	113 (77.9)	105 (77.8)	253 (80.1)	0.80
Chronic kidney disease, n (%)	37 (27.4)	37 (30.1)	72 (24.8)	0.53
Cancer, n (%)	30 (20.7)	25 (18.5)	65 (20.6)	0.95
PCS	45.7 (9.0)	44.7 (10.0)	36.1 (9.6)	< 0.0001
MCS	55.0 (8.2)	55.7 (8.1)	57.9 (8.2)	0.0004
Physical function	45.1 (10.7)	44.2 (11.0)	37.0 (10.4)	< 0.0001
Role physical	48.9 (8.9)	48.2 (9.2)	41.9 (9.7)	< 0.0001
Bodily pain	45.8 (9.9)	44.7 (11.4)	37.5 (10.2)	< 0.0001
General health	51.0 (7.8)	51.2 (8.9)	47.8 (9.4)	0.0001
Vitality	53.1 (9.5)	54.5 (9.3)	50.7 (9.1)	0.0002
Social functioning	52.9 (7.6)	52.3 (8.6)	50.8 (9.3)	0.03
Role emotional	50.5 (8.7)	50.3 (8.4)	50.0 (9.1)	0.81
Mental health	53.2 (8.4)	53.8 (9.1)	54.8 (7.8)	0.15
Gait speed, m/sec	0.98 (0.21)	0.97 (0.23)	0.97 (0.21)	0.92

Data presented as mean (standard deviation) for continuous variables, and no (%) for categorical variables

*PCS* Physical component summary score of SF-12, *MCS* Mental component summary score of SF-12

## Discussion

In a large cohort of healthy, community-dwelling older Australians, although participants receiving knee replacement experienced significant improvement in PCS score, their postoperative overall physical functional status remained significantly lower than for age- and sex-matched controls. The greatest improvements were observed for bodily pain and physical function, as expected after knee replacement surgery. Knee replacement had a modest negative impact on mental function. Worse preoperative physical function was the major predictor of minimal important improvement in PCS score following knee replacement. These findings suggest that the level of physical function is important for identifying older adults who have the greatest capacity to benefit from knee replacement surgery. Those who are already functioning well are less likely to benefit from this major surgery, and the risk–benefit ratio should be carefully considered.

There are limited data about the effect of knee replacement on functional outcomes in community-based older populations. Much of the available data are subject to bias conferred by population selection, such as samples drawn from hospital settings with single or multiple healthcare provider sites [3–7] or from clinical trials with strict eligibility criteria and standardised post-operative care and rehabilitation protocols [29]. In our study, participants received knee replacement as part of routine healthcare and decision making rather than being restricted by trial procedures or selection bias, reflecting the real-world situation. Furthermore, provision of knee replacement spanned both public and private health systems and importantly, was not restricted to single clinical sites. This may explain the higher pre-surgery PCS scores in our study compared with the previous studies [3–7], emphasising that highly-selected study populations and clinical samples of people undergoing knee replacement do not represent average community-dwelling older individuals.

As older people tend to experience decline in physical function related to ageing [16, 17], the impact of knee replacement on functional outcomes needs to be considered in this context. In the current study we were able to compare functional outcomes in participants following knee replacement with age- and sex-matched controls who had a similar likelihood of age-related decline over time. This is not possible for large-scale arthroplasty registry studies given the absence of a comparator group that does not receive surgery, nor hospital-based clinical studies where the focus is on postoperative improvement. Previous studies, using propensity score matching, reported improved physical functional status in older persons receiving TKR compared with controls without the surgery [30–32]. In our study, as expected, and supporting the findings from a previous nested cases-control study [33], participants with knee replacement had significantly worse PCS score and gait speed pre-knee replacement compared with matched controls; this likely reflects the indications for and decision to undergo surgery. However, although participants experienced improved PCS score after knee replacement, their PCS score remained significantly lower than for age- and sex-matched controls, with approximately half the pre-surgery differences persisting which was clinically significant [28]. In terms of the SF-12 health domains, bodily pain and physical function showed the most prominent improvements, with 12% and 8% improvement of their preoperative scores, respectively. These results suggest that knee replacement might improve physical function mainly by reducing pain. Although our study was unable to examine joint-specific pain, we found that knee replacement produced significant benefits through reducing bodily pain. With both participants with knee replacement and controls experiencing clinically nonsignificant decline in gait speed of a similar magnitude, gait speed remained significantly lower in participants following knee replacement than in age- and sex-matched controls.

In line with previous studies [34, 35], our study showed associations between worse preoperative physical function and minimal important functional improvement after knee replacement, as well as an association between better preoperative mental health and minimal important functional improvement after knee replacement. These data highlight the need for careful pre-surgery selection of patients based on their physical and mental health to identify those most likely to benefit from knee replacement surgery. Although knee arthritis is common in older adults, this does not mean the individual inevitably has significant functional impairment, as seen in our study. As knee replacements are aimed at improving physical function, the degree to which knee arthritis limits physical function would be a key criterion to determine the potential for benefit from this major surgery [34, 35]. Despite knee arthritis, the individual’s function might be limited by other joint disease or comorbidities [36, 37]. It may be supposed that mental health is adversely affected by severe arthritis and associated symptoms. However, we did not find that pre-surgery MCS score was significantly lower in participants with knee replacement than controls, rather there was a tendency to the converse with a reduction in MCS score post-knee replacement such that MCS score was similar in participants post-knee replacement and controls. We found that 24% of participants with knee replacement had worsened PCS score postoperatively, consistent with previous findings that about one quarter of individuals with technically successful TKR are dissatisfied with the outcomes [9, 10].

A strength of our study is the large community-dwelling, well-characterised cohort from which the study sample was drawn. The primary outcome of the ASPREE trial was disability-free survival, thus inclusion and exclusion criteria resulted in the cohort being free of chronic disability at enrolment. The approach of comparing participants with knee replacement with age- and sex-matched controls embedded in a large community-based clinical trial of older adults enables the investigation of knee replacement outcomes in the context of an ageing population with similar likelihood of age-related functional decline over time. The PCS and MCS scores in our study sample were comparable to the wider ASPREE cohort in which PCS scores declined while MCS scores tended to be stable with increasing age [38]. Being a trial conducted across multiple Australian states, knee replacements were undertaken across major public and private hospitals, and participants and their healthcare providers made decisions about knee replacement. There are limitations in our study. While 80.6% of the participants had completed SF-12 data, age, sex, baseline BMI, PCS, MCS, gait speed, and comorbidities were not significantly different between participants with completed data and those without. The time between baseline and follow-up outcome measures was longer for participants with knee replacement than for controls. We adjusted for this in the analyses and the results did not change compared with the results from unadjusted analyses. Joint-specific data were not collected in the ASPREE trial as the study focused on overall measures of wellbeing. We were unable to determine the type of knee replacement which could have been primary or revision knee replacement. Sensitivity analysis excluding participants with self-reported pre-trial joint replacement did not change the results. As voluntary participants in a long-term clinical trial, the ASPREE cohort may have greater interest in their health.

## Conclusions

In this community-based cohort of older adults, although those having knee replacement experienced significant improvements in PCS score, their postoperative overall physical functional status remained significantly lower than for age- and sex-matched controls. The greatest improvement was observed for bodily pain and physical function. The degree of preoperative physical function impairment was a strong predictor of functional improvement after knee replacement, suggesting that this could be an important consideration when identifying older people most likely to clinically benefit from knee replacement surgery. Care is needed in recommending knee replacement surgery for older adults who have good physical function, as they are less likely to benefit from this major surgery. The timing and risk–benefit ratio of knee replacement need to be carefully considered in older adults.

## Supplementary Information

**Additional file 1: Supplementary Figure 1.** Baseline and follow-up measures of self-reported physical and mental function in participants with knee replacement and age- and sex-matched controls, data presented as mean (standard deviation). **Supplementary Table 1.** Baseline and follow-up measures of health status and gait speed by gender in participants with knee replacement and age- and sex-matched controls. **Supplementary Table 2.** Changes in health status and gait speed from baseline to follow-up by gender in participants with knee replacement and age- and sex-matched controls. **Supplementary Table 3.** Baseline, follow-up, and changes in health status and gait speed in participants with knee replacement and age- and sex-matched controls excluding participants with self-reported joint replacement prior to ASPREE trial.

## Data Availability

The data generated from this study will not be deposited in a public repository due to privacy and consent restrictions. De-identified, coded data can be made available from the corresponding author upon reasonable request, subject to a data sharing agreement.

## Abbreviations

ANCOVA: Analysis of covariance
ANOVA: Analysis of variance
ASPREE: ASPirin in Reducing Events in the Elderly
BMI: Body mass index
CI: Confidence interval
MCS: Mental component summary
PCS: Physical component summary
SD: Standard deviation
SF-12: Medical Outcomes Study Short Form 12 Health Survey
TKR: Total knee replacement
