# Supplementary material for: Functional outcomes following knee replacement in community-dwelling older adults
Source: BMC Geriatr. 2023 May 2;23:261. doi: 10.1186/s12877-023-03925-y (PMC10152584; doi:10.1186/s12877-023-03925-y)
Supplement: Supplementary file 1 — Additional file 1: Supplementary Figure 1. Baseline and follow-up measures of self-reported physical and mental function in participants with knee replacement and age- and sex-matched controls, data presented as mean (standard deviation). Supplementary Table 1. Baseline and follow-up measures of health status and gait speed by gender in participants with knee replacement and age- and sex-matched controls. Supplementary Table 2. Changes in health status and gait speed from baseline to follow-up by gender in participants with knee replacement and age- and sex-matched controls. Supplementary Table 3. Baseline, follow-up, and changes in health status and gait speed in participants with knee replacement and age- and sex-matched controls excluding participants with self-reported joint replacement prior to ASPREE trial. [file 12877_2023_3925_MOESM1_ESM.pdf]

Supplementary Figure 1. Baseline and follow-up measures of self-reported physical and mental function in participants with knee replacement and age- and sex-matched controls, data presented as mean (standard deviation)

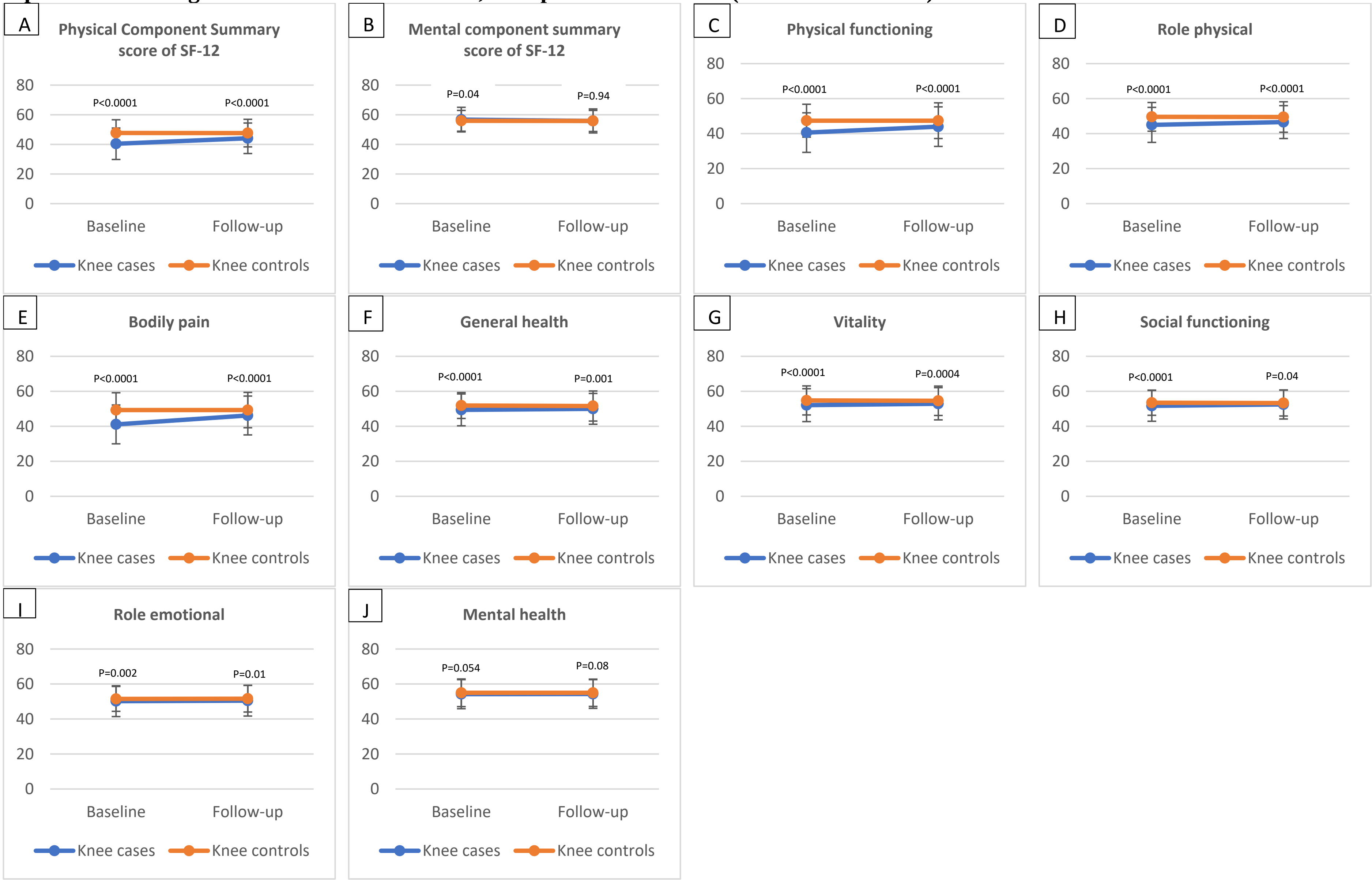

Supplementary Table 1. Baseline and follow-up measures of health status and gait speed by gender in participants with knee replacement and age- and sex-matched controls

	**Participants with knee replacement**			**Age- and sex-matched controls**			**Difference between groups**		
	Baseline Mean (SD)	Follow-up Mean (SD)	Change Mean (SD)	Baseline Mean (SD)	Follow-up Mean (SD)	Change Mean (SD)	Baseline P[c]	Follow-up P[c]	Change P[c]
**Females**	*n=349*			*n=502*					
PCS	38.8 (10.7)	42.0 (10.6)	3.2 (10.3)	46.8 (9.3)	46.7 (9.8)	-0.1 (7.8)	<0.0001	<0.0001	<0.0001
MCS	56.5 (8.4)	55.7 (8.5)	-0.8 (8.9)	55.5 (7.3)	55.4 (7.5)	-0.2 (7.5)	0.07	0.49	0.27
Physical function	38.7 (11.2)	41.3 (11.7)	2.5 (11.5)	45.8 (9.7)	46.0 (10.7)	0.1 (9.1)	<0.0001	<0.0001	0.001
Role physical	43.6 (10.1)	45.7 (9.5)	2.1 (10.7)	48.9 (8.5)	49.0 (8.9)	0.01 (8.9)	<0.0001	<0.0001	0.002
Bodily pain	40.2 (11.4)	44.6 (11.5)	4.4 (12.0)	48.5 (10.2)	48.5 (10.3)	0.0 (10.4)	<0.0001	<0.0001	<0.0001
General health	48.4 (9.0)	49.0 (9.1)	0.5 (8.6)	51.9 (7.6)	51.2 (8.6)	-0.6 (6.9)	<0.0001	0.0003	0.03
Vitality	50.8 (9.4)	51.4 (9.5)	0.6 (9.7)	54.0 (8.6)	53.6 (8.8)	-0.4 (8.8)	<0.0001	0.001	0.10
Social functioning	51.6 (8.7)	52.0 (9.1)	0.3 (10.3)	53.2 (7.7)	52.8 (7.9)	-0.3 (8.9)	0.007	0.15	0.30
Role emotional	49.4 (9.4)	50.3 (9.1)	0.9 (10.6)	51.0 (7.5)	51.4 (7.8)	0.4 (7.5)	0.006	0.054	0.43
Mental health	53.7 (8.0)	53.4 (8.5)	-0.3 (8.2)	54.3 (7.7)	54.0 (8.2)	-0.3 (8.2)	0.26	0.29	0.99
Gait speed (m/sec)[a]	0.94 (0.21)	0.89 (0.21)	-0.05 (0.18)	1.01 (0.22)	0.97 (0.22)	-0.05 (0.18)	<0.0001	<0.0001	0.97
**Males**	*n=247*			*n=310*					
PCS	42.7 (10.0)	46.9 (8.9)	4.3 (9.9)	49.2 (7.9)	49.1 (8.3)	-0.1 (7.1)	<0.0001	0.003	<0.0001
MCS	57.0 (8.0)	56.0 (7.4)	-1.0 (7.9)	56.4 (6.4)	56.5 (6.2)	0.1 (6.3)	0.36	0.34	0.06
Physical function	43.3 (10.9)	47.9 (9.4)	4.6 (11.1)	49.9 (8.4)	49.9 (9.1)	-0.03 (8.2)	<0.0001	0.01	<0.0001
Role physical	47.1 (9.4)	47.9 (9.1)	0.9 (10.4)	50.6 (7.6)	50.4 (8.2)	-0.2 (7.7)	<0.0001	0.001	0.16
Bodily pain	42.4 (10.6)	48.3 (10.1)	5.9 (11.6)	50.7 (9.3)	50.5 (9.6)	-0.2 (10.5)	<0.0001	0.01	<0.0001
General health	50.7 (8.9)	51.4 (8.3)	0.7 (7.9)	52.0 (7.2)	52.2 (8.5)	0.1 (7.7)	0.05	0.27	0.41
Vitality	54.0 (9.1)	55.0 (8.3)	1.0 (9.0)	56.1 (7.5)	56.1 (7.6)	0.0 (7.9)	0.002	0.09	0.17
Social functioning	51.7 (9.0)	53.2 (7.1)	1.5 (9.8)	54.1 (6.4)	54.2 (6.5)	0.1 (7.3)	0.0003	0.10	0.046
Role emotional	51.3 (7.9)	50.9 (8.4)	-0.4 (9.1)	52.4 (6.3)	52.0 (7.2)	-0.4 (7.0)	0.09	0.11	0.98
Mental health	54.8 (8.7)	55.4 (7.7)	0.6 (8.1)	56.1 (7.9)	56.6 (6.9)	0.5 (7.5)	0.07	0.06	0.83
Gait speed (m/sec)[b]	1.02 (0.20)	1.00 (0.18)	-0.02 (0.19)	1.09 (0.22)	1.03 (0.21)	-0.06 (0.18)	0.001	0.054	0.07

SD: standard deviation; PCS: Physical component summary score of SF-12; MCS: Mental component summary score of SF-12

[a]n=262 for female participants with knee replacement and n=463 for female controls

[b]n=193 for male participants with knee replacement and n=283 for male controls

[c]For difference between participants with knee replacement and controls in each of the baseline and follow-up measures and their change from baseline to follow-up

Supplementary Table 2. Changes in health status and gait speed from baseline to follow-up by gender in participants with knee replacement and age- and sex-matched controls

	Participants with knee replacement		Age- and sex-matched controls		Difference between groups	
	Mean change (95% CI)	P	Mean change (95% CI)	P	Mean difference (95% CI)	P
**Females**	*n=341*		*n=500*			
PCS	3.1 (2.2, 4.1)	<0.001	-0.03 (-0.8, 0.8)	0.93	3.2 (1.9, 4.4)	<0.001
MCS	-0.9 (-1.8, -0.1)	0.03	-0.03 (-0.7, 0.7)	0.94	-0.9 (-2.1, 0.2)	0.12
Physical function	2.4 (1.3, 3.5)	<0.001	0.3 (-0.6, 1.2)	0.55	2.2 (0.7, 3.6)	0.003
Role physical	1.9 (0.8, 2.9)	<0.001	0.2 (-0.7, 1.0)	0.67	1.7 (0.3, 3.1)	0.02
Bodily pain	4.2 (3.0, 5.4)	<0.001	0.1 (-0.8, 1.1)	0.78	4.0 (2.5, 5.6)	<0.001
General health	0.6 (-0.2, 1.5)	0.13	-0.7 (-1.4, -0.1)	0.03	1.4 (0.3, 2.5)	0.01
Vitality	0.4 (-0.6, 1.4)	0.44	-0.2 (-1.0, 0.6)	0.62	0.6 (-0.7, 1.9)	0.37
Social functioning	0.1 (-1.0, 1.1)	0.92	-0.2 (-1.1, 0.6)	0.61	0.3 (-1.1, 1.6)	0.69
Role emotional	0.8 (-0.2, 1.7)	0.11	0.6 (-0.2, 1.4)	0.15	0.2 (-1.1, 1.5)	0.75
Mental health	-0.4 (-1.3, 0.5)	0.40	-0.2 (-0.9, 0.6)	0.64	-0.2 (-1.4, 1.0)	0.73
Gait speed (m/sec)[a]	-0.05 (-0.07, -0.03)	<0.001	-0.05 (-0.06, -0.03)	<0.001	-0.004 (-0.03, 0.02)	0.77
**Males**	*n=240*		*n=308*			
PCS	4.2 (3.1, 5.3)	<0.001	-0.01 (-1.0, 0.9)	0.99	4.2 (2.8, 5.7)	<0.001
MCS	-1.0 (-1.9, -0.1)	0.03	0.1 (-0.7, 0.9)	0.80	-1.1 (-2.3, 0.1)	0.07
Physical function	4.7 (3.5, 5.9)	<0.001	0.2 (-0.9, 1.3)	0.72	4.5 (2.8, 6.1)	<0.001
Role physical	0.7 (-0.4, 1.9)	0.22	-0.1 (-1.1, 0.9)	0.86	0.8 (-0.7, 2.4)	0.30
Bodily pain	5.8 (4.4, 7.2)	<0.001	-0.3 (-1.5, 1.0)	0.68	6.0 (4.2, 7.9)	<0.001
General health	0.7 (-0.3, 1.7)	0.18	0.3 (-0.6, 1.2)	0.52	0.4 (-0.9, 1.7)	0.56
Vitality	0.9 (-0.1, 2.0)	0.09	0.1 (-0.9, 1.0)	0.84	0.8 (-0.6, 2.3)	0.26
Social functioning	1.7 (0.7, 2.8)	0.001	-0.05 (-1.0, 0.9)	0.92	1.8 (0.4, 3.2)	0.02
Role emotional	-0.5 (-1.5, 0.5)	0.33	-0.3 (-1.2, 0.6)	0.51	-0.2 (-1.6, 1.2)	0.77
Mental health	0.5 (-0.5, 1.5)	0.30	0.5 (-0.4, 1.4)	0.27	0.04 (-1.3, 1.4)	0.96
Gait speed (m/sec)[b]	-0.02 (-0.05, 0.004)	0.09	-0.06 (-0.08, -0.03)	<0.001	0.03 (-0.002, 0.07)	0.07

CI: confidence interval; PCS: Physical component summary score of SF-12; MCS: Mental component summary score of SF-12

All analyses adjusted for baseline body mass index and days between outcome measures

[a]n=256 for female participants with knee replacement and n=461 for female controls

[b]n=190 for male participants with knee replacement and n=283 for male controls

Supplementary Table 3. Baseline, follow-up, and changes in health status and gait speed in participants with knee replacement and age- and sex-matched controls excluding participants with self-reported joint replacement prior to ASPREE trial

	**Participants with knee replacement N=358**			**Age- and sex-matched controls N=699**			**Difference between groups**	
	Baseline Mean (SD)	Follow-up Mean (SD)	Change Mean (95% CI)[¶]	Baseline Mean (SD)	Follow-up Mean (SD)	Change Mean (95% CI)[¶]	Change Mean (95% CI)[¶]	P[¶]
PCS	40.8 (10.6)	44.9 (9.9)	3.9 (3.0, 4.8)	48.4 (8.6)	48.2 (9.1)	0.003 (-0.6, 0.6)	3.9 (2.7, 5.0)	<0.001
MCS	57.1 (8.2)	55.8 (8.0)	-1.4 (-2.2, -0.6)	55.7 (6.9)	55.6 (7.2)	-0.1 (-0.6, 0.5)	-1.3 (-2.3, -0.4)	0.01
Physical function	40.9 (11.1)	44.4 (11.2)	3.4 (2.4, 4.5)	48.0 (9.2)	48.2 (9.9)	0.4 (-0.4, 1.1)	3.1 (1.8, 4.4)	<0.001
Role physical	45.9 (9.9)	47.1 (9.3)	1.0 (-0.03, 2.0)	50.0 (8.1)	49.9 (8.5)	-0.01 (-0.7, 0.7)	1.0 (-0.3, 2.2)	0.12
Bodily pain	41.4 (11.2)	47.5 (10.6)	5.7 (4.6, 6.9)	49.8 (9.7)	49.8 (10.0)	-0.002 (-0.8, 0.8)	5.7 (4.3, 7.1)	<0.001
General health	49.6 (9.0)	50.4 (8.3)	0.9 (0.03, 1.7)	52.1 (7.4)	51.7 (8.7)	-0.4 (-0.9, 0.2)	1.2 (0.2, 2.2)	0.02
Vitality	53.1 (9.1)	53.1 (9.1)	-0.2 (-1.2, 0.7)	55.1 (8.2)	54.9 (8.3)	-0.1 (-0.8, 0.5)	-0.1 (-1.3, 1.0)	0.85
Social functioning	52.0 (8.8)	52.8 (8.0)	0.8 (-0.1, 1.8)	53.7 (7.0)	53.3 (7.5)	-0.4 (-1.0, 0.3)	1.2 (0.1, 2.4)	0.04
Role emotional	50.5 (8.8)	50.3 (9.0)	-0.2 (-1.1, 0.6)	51.6 (7.0)	51.7 (7.6)	0.2 (-0.4, 0.8)	-0.4 (-1.5, 0.7)	0.46
Mental health	54.5 (8.1)	54.6 (8.2)	-0.05 (-0.9, 0.8)	54.9 (8.0)	55.0 (8.0)	0.1 (-0.5, 0.7)	-0.1 (-1.2, 1.0)	0.81
Gait speed (m/sec)*	0.96 (0.21)	0.93 (0.20)	-0.04 (-0.06, -0.01)	1.05 (0.22)	1.00 (0.22)	-0.05 (-0.07, -0.04)	0.02 (-0.01, 0.04)	0.28

SD: standard deviation; CI: confidence interval; PCS: Physical component summary score of SF-12; MCS: Mental component summary score of SF-12

*n=260 for participants with knee replacement and n=641 for controls

[¶]Adjusted for baseline body mass index and days between outcome measures
